# The Experience of Physical Recovery and Physical Rehabilitation Following Hospital Discharge for Intensive Care Survivors—A Qualitative Systematic Review

**DOI:** 10.3390/nursrep14010013

**Published:** 2024-01-09

**Authors:** Sian Goddard, Hilary Gunn, Bridie Kent, Rachel Dennett

**Affiliations:** 1 Faculty of Health, School of Health Professions, University of Plymouth, Plymouth PL4 6AB, UK; 2 Faculty of Health, School of Nursing and Midwifery, University of Plymouth, Plymouth PL4 8AA, UK

**Keywords:** rehabilitation, intensive care, critical illness

## Abstract

Background: Over 120,000 people in the UK survive critical illness each year, with over 60% of these experiencing mobility issues and reduced health-related quality of life after discharge home. This qualitative systematic review aimed to explore critical care survivors’ perceptions, opinions, and experiences of physical recovery and physical rehabilitation following hospital discharge. Methods: This review followed the Joanna Briggs Institute (JBI) methodology with the Preferred Reporting Items for Systematic Reviews and Meta-Analyses (PRISMA) and was conducted between January 2020 and June 2022. The search was conducted using the following databases: Embase, CINAHL, Medline Ovid, Cochrane, and the Joanna Briggs Institute, and sources of grey literature were searched for eligible studies. Qualitative studies focused on physical rehabilitation or recovery, involving adult survivors of critical illness who had been discharged from hospital. Results: A total of 7 of 548 identified studies published in 2007–2019 were eligible for inclusion. The findings indicate that qualitative evidence around the experiences of physical recovery and rehabilitation interventions following discharge home after critical illness is limited. Three synthesised findings were identified: ‘Positivity, motivation and hope’; ‘Recovery is hard and patients need support’; and ‘Patients experience challenges in momentum of physical recovery’. Conclusions: Survivors struggle to access healthcare professionals and services following discharge home, which influences the momentum of physical recovery. Supervised exercise programmes had a positive impact on the perception of recovery and motivation. However, ‘simple’ structured exercise provision will not address the range of challenges experienced by ICU survivors. Whilst some factors influencing physical recovery are similar to other groups, there are unique issues experienced by those returning home after critical illness. Further research is needed to identify the support or interventions survivors feel would meet their needs and assist their physical recovery. This study was prospectively registered with Prospero on 3/2/2020 with registration number CRD42020165290.

## 1. Introduction

Critical care units across the UK treat over 161,000 patients every year, [1] with the median average intensive care unit (ICU) stay being 2.6 days and hospital stay being 19.3 days (although this can be months for many who require rehabilitation). The majority of these patients (79.6%) survive to hospital discharge; however, many report reduced health-related quality of life (HRQL) for months and even years after discharge.

Although some survivors have permanent restrictions, which limit their ability to return to pre-admission levels of activity and mobility, there is also a proportion who fail to achieve their rehabilitation potential as a result of limited rehabilitation support for physical, psychological, and cognitive issues. This is particularly seen in patients whose intensive care stay was longer than 2 days [2,3].

A multi-centre study by Griffith et al. [2] included a twelve-month follow-up period and 283 participants and identified significantly reduced HRQL measures in critical care survivors when compared with population norms. The study also demonstrated functional deficits: two-thirds reported walking problems six months after leaving hospital, and 44% were significantly anxious or depressed. These findings are supported by McNelly et al. [4], who identified reduced activity levels and HRQL in critical care survivors, which was compounded in those with pre-critical illness morbidities or frailty.

The effects of critical illness on physical function, skeletal muscle depletion, and health-related quality of life have been well documented since the identification of intensive-care-unit-acquired weakness [5], which results in a decline in muscle mass, muscle contractility and denervation, as well as bone density loss. Patients on bed rest are reported to lose up to 5.2% of their muscle mass in the first two weeks [6]. This is in addition to a 1% loss of bone density [7] and bone demineralisation [8] reported in immobilised healthy subjects.

The benefits of early mobility have been highlighted and well researched since the introduction of the NICE (National Institute for Health and Care Excellence) Guidelines for critical care rehabilitation published in 2009 [9]. In light of this evidence, significant improvements have been made in the treatment and management of patients in ICUs to minimise the impact of the effects of immobility. The NICE guidelines have led to significant improvements in the provision of therapy within the hospital setting, recommending the provision of follow-up services including rehabilitation. This has led to many small studies investigating rehabilitation following discharge.

However, the continuation of rehabilitation and provision of follow-up services is sporadic as a result of underfunding and a lack of conclusive evidence. This was initially identified by Connolly et al. in 2014 [10], and a follow-up study in 2021 still highlighted underfunding as an issue [11]. The updated survey by Connolly et al. published in 2021 [11] included representation from across UK regions and hospital specialities. It reported that only 12 out of 182 (6.8%) hospitals surveyed offered a post-hospital discharge rehabilitation service, with lack of funding cited as the main reason for this. This was highlighted earlier by a global consensus conference in 2012 [5], which identified an issue with funding for post-discharge rehabilitation because of a lack of robust clinical evidence in favour of particular rehabilitation programmes.

A Cochrane review of rehabilitation following intensive care unit discharge in 2015 [12] was unable to determine an overall result for the effects of exercise-based rehabilitation interventions. The authors highlighted significant methodological variation in the studies and identified a need for further research considering the experience and acceptance of exercise-based rehabilitation interventions.

Walker et al. [13] suggest that the lack of consideration of a patient’s experiences of exercise rehabilitation programmes following critical illness may explain the limited effects demonstrated in many of the quantitative studies. The paucity of qualitative evidence means there is also limited consideration of the patient’s perception of returning to physical activity. The importance of active engagement and ownership of the programme has been demonstrated to influence its success in other populations, such as the stroke survivor population, who traditionally have had a more formal rehabilitation process following hospital discharge. In a systematic review of patients’ experiences of rehabilitation following a stroke, Peoples, Satink, and Steultjens [14] found that participants identified a need for active participation in their rehabilitation. Additionally, they highlighted that factors such as a lack of information prevented them from feeling they were making progress and influenced their perceptions of recovery. The review identified strong themes, particularly the need for empowerment and how the lack of it influenced the engagement with, and outcomes of, rehabilitation.

Although this work has been carried out in the stroke, cardiac rehabilitation, and other chronic disease groups, none has been carried out in the critical illness survivor population. Parry et al. [15] identified, in a comprehensive review, that specific rehabilitation programmes were needed for this group, and they highlighted the benefits of physical activity. However, a lack of funding and provision and a lack of education on the part of healthcare professionals and caregivers was a barrier to engagement.

Ågård and Egerod [16] investigated the issues around the struggle for independence encountered by ICU survivors a year after their discharge home. The study participants reported a focus on regaining functional abilities in the year following discharge. This, again, suggests that understanding patients’ perceptions and experiences in the months following discharge would assist in the design and provision of meaningful and relevant rehabilitation interventions.

One mixed, quantitative, and qualitative systematic review was carried out by Parry and Knight [17], which covered both in-hospital and post-ICU settings. The wealth of reviewed literature in the study was about in-hospital barriers and enablers to rehabilitation and, therefore, was not focused on the experiences of physical activity, exercise, or physical rehabilitation in the intensive care survivor population following hospital discharge, which was the intended focus of this review.

There have been a number of qualitative studies considering patients’ perceptions of interventions post-hospital discharge; however, to date, there has not been a systematic review collating the findings and informing future practice.

A greater understanding of patients’ experiences of physical recovery and perceptions of exercise rehabilitation via a formal review of the literature is essential to ensure that patients’ perspectives can inform and influence the development of future rehabilitation services or guidance.

The aim of this review was to explore critical care survivors’ perceptions, opinions, and experiences of physical recovery and physical rehabilitation (in any form) following hospital discharge.

## 2. Materials and Methods

The study objectives identified in the study protocol were (1) to identify positive and negative aspects of physical activity or physical rehabilitation interventions from the patient’s perspective; (2) to identify experiences of aspects of rehabilitation interventions such as the location, content, and frequency of service provision; and (3) to gain an understanding of the issues that most influence patients’ experiences of physical recovery, either positively or negatively.

To provide a comprehensive, unbiased synthesis of the existing knowledge, this systematic review was conducted in accordance with the JBI methodology for systematic reviews of qualitative evidence. The systematic review protocol was registered with the International Prospective Register of Systematic Reviews (PROSPERO CRD42020165290). This review is reported in accordance with the PRISMA guidelines [18].

### 2.1. Participants, Phenomenon of Interest, and Context Framework

When devising the inclusion and exclusion criteria presented in Table 1, the PICo mnemonic for qualitative research was used to support and structure the questions. The core elements of PICo are outlined below and include P, indicating participants; I, indicating the phenomenon of interest; and Co, indicating the context [19]. The following definitions of physical recovery and physical rehabilitation were applied: Physical recovery refers to the progression and return of physical function and the ability to complete activities of daily living relevant to the individual’s previous activity levels. This does not include psychological recovery. Physical rehabilitation is the process of helping someone recover from injury or illness and regain their strength and mobility.

Phenomenon of interest: Studies investigating patients’ experiences or perceptions of physical activity or physical rehabilitation following discharge home after intensive care survival were considered in this review. This review did not limit the type of physical activity or physical rehabilitation intervention investigated in studies. A minor modification was made to the published protocol for the inclusion of studies to remove the requirement for studies to be focused on an intervention. This was carried out following the application of inclusion/exclusion criteria, which would have resulted in the inclusion of only two studies. Following the minor modification, qualitative studies that considered perceptions and experiences of physical recovery and physical rehabilitation as well as interventions were included.

Context: Studies that were conducted among post-critical-care survivors who required or experienced rehabilitation in any setting(s), including at home, outpatient clinics, and community venues, were included. This review excluded any participants who were discussing rehabilitation undertaken whilst being an inpatient. This review included studies where the views of participants’ relatives were reflected in the findings since excluding these studies would have unduly limited the review. Relatives’ views identified in the included studies were only included in the review findings where they were specifically relevant to the critical care survivors’ perceptions, opinions, and experiences of physical recovery and physical rehabilitation.

Types of studies: Those focused on qualitative data including, but not limited to, designs such as phenomenology, grounded theory, ethnography, action research, and feminist research were included. International studies published in English were considered for inclusion in this review where the full text was available. No date limits were set for the database searches.

### 2.2. Search Strategy

A three-step search strategy was utilised identifying both published and unpublished studies.

Following an initial limited search of MEDLINE and CINAHL via Ovid to identify the text words contained in the titles and abstracts, and of the index terms used, a second search was undertaken across all included databases using the following terms: (Intensive Care OR critical care OR critical illness OR ICU), AND (Physical recovery OR exercise training OR exercise-rehabilitation OR rehabilitation OR follow-up rehabilitation OR physical activity) AND (Experience OR perception OR opinion OR attitude OR view OR qualitative OR feeling OR belief OR perspective).

The search strategy included the following databases: AMED, CINAHL, the Cochrane Central Register of Controlled Trials, EMBASE, the JBI Database of Systematic Reviews and Implementation Reports, and MEDLINE. The search for unpublished studies included ProQuest dissertations and theses, Open Grey, Google Scholar, and the Conference Papers Index. Finally, the reference list of all identified reports and articles included in the following full-text review was searched for additional studies.

### 2.3. Study Selection

The results of the database searches were imported to Mendeley Reference Manager for the removal of duplicates and title and abstract screening by the first author, then confirmed by the second author before the remaining studies were retrieved for full-text reviews.

The titles and abstracts of the selected papers were assessed by two independent reviewers against the inclusion criteria. Those meeting the inclusion criteria were retrieved in full, and the information was reported using the standardised critical appraisal instruments from the Joanna Briggs Institute System for the Unified Management, Assessment, and Review of Information [19]. Full-text studies that did not meet the inclusion criteria were excluded. The included studies were critically appraised by two independent reviewers.

### 2.4. Assessment of Methodological Quality

Full-text studies were critically appraised by two independent reviewers (S.G. and R.D.) for methodological quality using the standardised JBI critical appraisal checklist for qualitative research [20]. A consensus process was used to determine a study’s inclusion, with independent appraisal carried out by two members of the review team. There were no disagreements between the reviewers surrounding study inclusion, though a third reviewer was available should this have been required. Prior to this review, it was the reviewers’ intention that all studies, regardless of the methodological quality, should undergo data extraction and synthesis using meta-aggregation. The limited number of studies identified following the appraisal and application of the exclusion criteria meant that this decision with regard to quality was prudent.

### 2.5. Data Extraction

Qualitative data were extracted from papers included in this review using the standardised JBI qualitative data extraction tool [21]. Operational guidelines and definitions contained in the published JBI information regarding meta-aggregation were used to guide the data extracted. In meta-aggregation, data extraction occurred in two phases. Phase 1 involved details of the study populations, the context of physical rehabilitation intervention, culture, geographical location, study methods, and the phenomenon of interest. Following the minor modification, qualitative studies that considered perceptions and experiences of physical recovery and physical rehabilitation as well as interventions were included. Phase 2 included analytical data and an illustration of each finding from the included studies, which would be assigned a JBI level of credibility.

The data extracted included specific details about the interventions, populations, study methods, and outcomes of significance to the review question and specific objectives (Supplementary Material S1).

### 2.6. Data Synthesis

The qualitative research findings were aggregated using the meta-aggregation methodology of JBI [19] to identify categories and amalgamate existing qualitative findings around the experiences and perceptions of physical recovery or rehabilitation following discharge home after critical illness, in line with the objectives of this review. This involved a comprehensive, exhaustive search and independent critical appraisal including a standardised data extraction [19]. The extracted findings were then rated according to their level of credibility, i.e., unequivocal, credible, or not supported. Unequivocal (U) related to evidence beyond a reasonable doubt, which may include findings that are a matter of fact, directly reported/observed, and not open to challenge [20]. The synthesis of data did not include any “credible” or “not supported” findings.

The rated findings were categorised based on similarity in the meanings of ideas or concepts. One reviewer (S.G.) performed the data synthesis, which was checked by the second reviewer (R.D.) to confirm the credibility level. Direct participant quotes were identified and attached to each finding; therefore, all were assessed at the “unequivocal” level. Once findings had been assigned a level of credibility, they were then grouped and agreed upon by two reviewers. These categories were then subjected to a meta-aggregation to produce a single comprehensive set of synthesised findings that formed the results and related to the aims of this systematic review. These were agreed by all reviewers [19].

### 2.7. Assessing Certainty in the Findings and Strength of Evidence

The final synthesised findings were graded according to the ConQual approach [20] for establishing confidence in the output of qualitative research synthesis and are presented in the summary of the findings (Supplementary Material S2). The summary of the findings includes the major elements of this review and details on how the ConQual score is developed. Included in the table are the title, population, phenomenon of interest, and context for the specific review. Each synthesised finding from this review is presented along with the methodological approach informing it, a score for dependability, credibility, and the overall ConQual score.

## 3. Results

**Figure 1:**
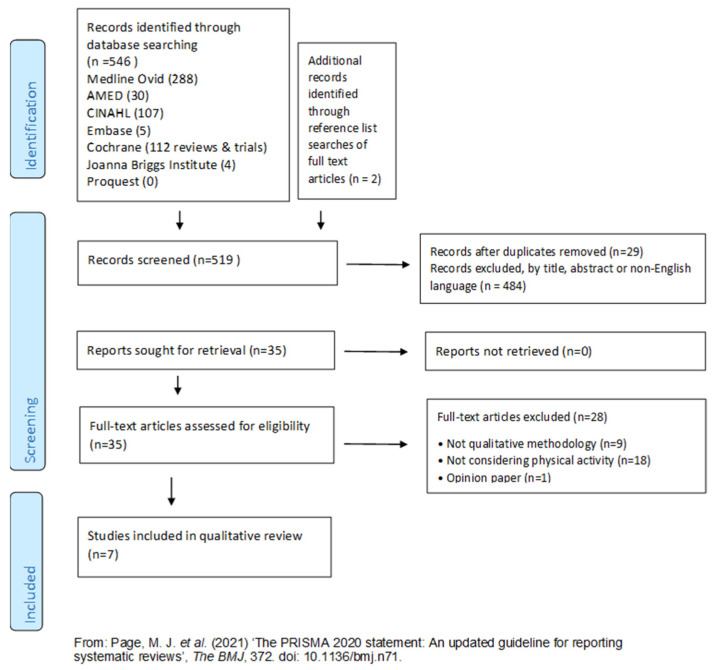
Preferred Reporting Items for Systematic Reviews and Meta-Analysis (PRISMA) flow diagram [18].

A total of 546 papers were identified via electronic databases (see PRISMA diagram in Figure 1). After 29 duplicates were removed and 484 studies were excluded, 35 full-text studies were included for eligibility assessment based on the inclusion criteria. Following full-text screening, seven were included. As the phenomenon of interest was the perceptions and experiences of critical care survivors, only primary sources where the participant voices of the ICU survivors or carers were selected.

### 3.1. Methodological Quality

The included studies were assessed to be of moderate–high methodological quality with scores of between 6/10 and 10/10 based on the ten questions of the JBI critical appraisal tool (Supplementary Material S3). The aims, objectives, and data collection method were congruent with a qualitative study design; thus, the reviewers could infer the qualitative nature of the design and respond affirmatively to Q2, Q3, Q4, and Q5. Aside from Q6 concerning the researcher’s cultural or theoretical background and Q7 concerning the influence of the researcher on the research, and vice-versa, the authors of the included studies responded adequately to the remaining questions.

### 3.2. Study Characteristics

Of the seven included studies, five used semi-structured interviews ([16,22,23,24]), one study used a qualitative online questionnaire [25], and one used focus groups [13].

The location of in-person interviews varied between studies. Three studies were based in the UK, two in the US, one in Denmark, and one in Canada, and the final study [25] included participants from the UK, the US, Australia, and Canada. Two studies by Walker and Wright [13,23] were qualitative evaluations of physical rehabilitation interventions.

The number of participants ranged from 5 to 35 with interviews or focus groups being carried out between 1 and 24 months post-discharge home. There were 120 participants across the included studies. The age range of participants was 18–97 years (The characteristics of the included studies can be seen in Supplementary Material S2). The participant numbers in the included studies were as follows: Agard et al., n = 18, Corner et al., n = 15; Czerwonka et al., n = 5; Deacon, n = 35; Ferguson et al., n = 21; Redwine, n = 10; and Walker et al., n = 16.

### 3.3. Review Findings

Forty-seven findings were identified from the seven included studies. All findings were derived from unequivocal data, which were supported by the participant voices in the studies.

**Figure 2:**
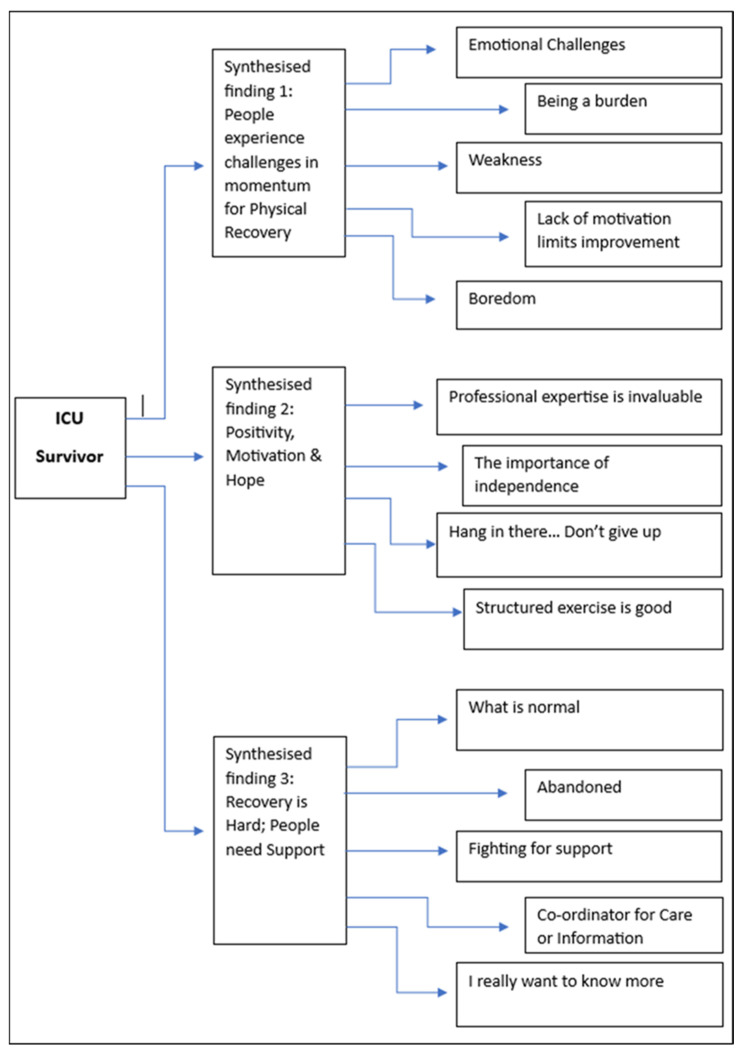
Synthesised findings and categories.

Three synthesised findings arose from the analysis and categories within the seven studies. The illustrated links between the categories and synthesised findings can be seen in Figure 2. (The meta-aggregated flow charts for each synthesised finding, with detailed links between the synthesised findings, their related categories, and the findings are available online in Supplementary Material S4).

### 3.4. Synthesised Finding 1: People Experience Challenges in Momentum of Physical Recovery

This synthesised finding (Figure 2) arose from the identification of physical, emotional, and psychological factors that affected the trajectory of physical recovery. The categories and findings arose from participants in six of the seven studies within this review. The synthesised findings identified issues around a lack of motivation limiting improvement, alongside emotional challenges leaving participants feeling vulnerable and withdrawn; feelings of being a burden as a result of a lack of physical ability alongside the effects of physical weakness; and, finally, boredom.

Emotional challenges concerning recovery, interventions, and rehabilitation were the most clear category arising from the findings. Participants described being scared or feeling vulnerable: “to have moments on my own… it’s just a bit scary… you know if something happens and there’s nobody to sort of ask”; “I was scared. I never used to be frightened of nothing” [13].

Descriptions of the burden of emotional load were also an issue: “I got very down, depressed, halfway through the programme” and “it was hard to judge the effect of it… Because I sort of became withdrawn… the mental effects overweighed the physical” [23].

This was supported by participants who identified that the physical changes and reduced physical ability led to emotional issues: “ it’s a bit degrading that you can’t do what you could do”; “I’ve got a lot of anger because I’ve got facial deformities” [13]. In addition to this, participants in one study described the frustration of social withdrawal as a result of critical illness—“I don’t socialise as much as I used to”; “if you go out with friends… they don’t want to be talking about your illness” [13]—and linked this to the frustration of realising they could not return to previous activities: “I got a lot of work to do around here… I ain’t moved that truck since you’ve been here, neither one of ‘em. I hadn’t been down there a fishing, or a going anywhere. Don’t feel like it” [24].

Alongside emotional barriers to recovery, weakness was a theme frequently identified across studies. The persistence of weakness and the demoralising effects of it were tied in with the aforementioned emotional challenges.

In one study, participants spoke of the impact of ongoing physical effects: “I can now walk with two sticks… but that’s after two years”; “I was a mechanic…. I can’t do that now. I’m not allowed to drive a car or get on a plane; they won’t let me do anything” [13]. And in another study, participants stated “I couldn’t even hold my head. I wasn’t able to do anything” and “I felt it took forever before I regained my strength…. I don’t feel I am up to my usual strength yet” [16].This was supported by contrasts with findings from the study by Corner et al. [22], where the idea of a recalibration of the self was identified: “I didn’t realise I couldn’t walk. I thought I could and I tried load of times… I was weak… I couldn’t do it” [22]. Participants’ comments expressed that the persistence of physical weakness required them to adjust their perception of their abilities and recovery timeframes.

A further category arising from the findings was a lack of motivation. This was clearly a barrier to recovery for participants in some studies. Some described a temptation to think “oh do I do something else or watch the telly” [13]. This ran alongside the emotional effects on motivation: “I got very down, depressed” and “the mental effects overweighed the physical” [23].

Some described a lack of time [23] and ongoing fatigue affecting participants’ motivation to progress. Although for some participants, time was not an issue, for others, the need to return to work led to the acceptance of reduced physical recovery.

Finally, the negative effect of boredom in the context of rehabilitation and physical activity was discussed by participants: “that’s the worst things about coming out of hospital, sitting doing nothing” or “just bored all the time” were comments in one study, alongside and different to “being at home relatively locked up” [13]. These findings indicate the complex interaction between physical and emotional difficulties and the way in which they may impact the recovery process.

### 3.5. Synthesised Finding 2: Positivity, Motivation, and Hope

The second synthesised finding conveyed the positive aspects of physical activity, motivation and hope surrounding recovery and rehabilitation. The meta-aggregated flow chart showing the relationship between findings, categories, and the synthesised finding of “Positivity, Motivation and Hope” can be seen in Figure 2.

This synthesised finding comprised four categories.

The strongest influence on motivation was categorised as “professional expertise is invaluable”. The benefit of expertise from healthcare professionals enabled participants to feel supported, safe, and confident to engage in more demanding physical exercise: “to have [the physiotherapist] explaining everything to me, making sure I knew then I could trust that if she was pushing, I felt safe with her” [23] “so they tailored it to my needs” [23], and “he’s always willing to sit down with you and talk with you about your condition” [24].

Participants valued the reassurance, encouragement, and caring approach offered by healthcare professionals: “sometimes I think, I feel like I am [overdoing it], but the doctors says it’s alright” [24] and “look after you”, “one to one… just focusing on you” [13].

The second category within this synthesised finding was identified as “the importance of independence”, specifically, the desire for independence to drive the progression of recovery.

In one study, participants reported the joy of regaining functional capacity, “happiness is doing things yourself” [16], and in another, “just being able to do little things for myself” [26].

Having a positive outlook, perseverance, and an intrinsic desire for independence were also identified as factors: “been at deaths door and then… being given a second chance” [13] and “keep going”, “hang in there”, “be patient”, and “don’t give up” [16].

Some participants discussed the adaptations they had instigated to allow independence: “in the beginning when I came home and wanted to go upstairs, I sat on my behind and went up and down the stairs. It took a while before I could get around” [23].

The recognition of the value of modifying aspects of life to facilitate independence contrasted with those for whom independence remained an aspiration: “I probably went too far… tried to arrange that my husband didn’t need to come home and do things”, or “when I can walk again, it will be different” [23].

Participants also discussed the transition from dependence to being more independent: “I didn’t have the strength to do it, but I had to… because I wasn’t given the option”; “it’s great in a way because you have to do things on your own. And then you start getting stronger and take rest breaks and you do it again” [26].

In conjunction with the motivating effect of independence, other findings were summarised in the category, “Hang in there… don’t give up”. The need for perseverance and training reflected an underlying positivity towards recovery: “keep going”, “hang in there”, and “don’t give up” [23]. This sat alongside the motivation and positivity gained from the recognition of milestones towards recovery: “I was shocked at how little I could do but now, it’s the other way, I’m actually shocked at how much I can do and am doing” [22].

Finally, some participants gained hope from comparing themselves with others: “you got people’s in worser shape than I am”; “I think I’ll never be able to do maybe what a lot of people do, but I think that I can at least live what I would call to be, a normal life” [24].

As part of the synthesised finding “Positivity, Motivation and Hope”, many of the findings focused on the positivity and motivating influence of structured exercise. This was drawn together in the category “structured exercise is good”.

Most of the findings originated from two studies, namely, the Revive Trial [23] and PIX [13] studies.

Participants clearly endorsed the benefits of an exercise programme: “It’s just vital”, “I don’t know how I would have managed without it”, “something to look forward to”, and “an incentive to recover” [23].

Separately from another study, participants identified the joy in being able to participate in structured exercise: “I really enjoyed it” and “I love it all, every bit of it” [13].

Aside from these endorsements, there were comments on the physical benefits, which participants extended into their activities of daily living: “you can end up doing nothing for months so yeah…. It was very good for that side, it actually gets you to a point that you did more” [13]; “if I hadn’t come in for these six weeks of the trial, I’d still have been struggling to get off a chair” [23]; and “a sense of achievement every time you went” or “you felt like you were progressing” [13].

The latter comments also endorsed the psychological effects mentioned by participants: “it sort of improved your mind a lot as well”; “it gave you space to think, gave your brain a break, instead of being sat at home thinking about it constantly” [13].

This ran alongside comments relating to the challenge of exercising alone, which was felt to be less motivating than in a group, where the benefit of a programme with others was clear: “be in a class with people like myself” or “being forced into motivation and forced into fitness” [13].

### 3.6. Synthesised Finding 3: Recovery Is Hard and Patients Need Support

The final theme that was evident from the findings and categories was that of the challenges experienced and the need for support. Across this theme, the strength of feeling was evident in the findings, with anger, frustration and desperation expressed as barriers to recovery and rehabilitation, as presented in synthesised finding 1 (people experience challenges in momentum for physical recovery).

The meta-aggregated flow chart showing the relationships between the findings, categories, and synthesised findings can be seen in Figure 2.

Having an understanding of what to expect in terms of physical recovery was identified as being an important factor. This was expressed by one participant, as a “need to know what is normal” [25].

This was also mentioned in the same study by a separate participant who explained the need to know “what to expect in the future. Knowing what is normal relieves stress” [25]. The stress of not knowing what was supposed to happen and the difficulty of being asked for their input despite limited knowledge, was echoed separately in the comments “they did not know what goals to set” and it was “like being in a car crash and someone asking you how you want to be cut out” [22]. Continuity of care was seen as important in helping participants understand what is normal during recovery: “I think I probably would have liked to receive more contact with the healthcare system, because you’re not quite sure how your recovery is going” [26] or “right now, I would like to know more about what happened… you know my recovery time, how come it’s taking so long, and I really want to know more” [26], and finally, “two week delay for his rehabilitation to start… two weeks after an ICU stay for a survivors is a long, long time” [26].

In some findings, the difficulties associated with recovery were expressed as abandonment and being kept in the dark. Participants in one study expressed, “you just feel like you’re out on a limb all of a sudden”, while another stated, “dumped at home with two sticks…I was just a number then”, and a third explained, “it felt like they just wanted a bed and had to throw me out… I just felt like… sort of abandoned really” [13].

Linked to the expressions of need for care when feeling abandoned, was the category, Fighting for Support. Participants expressed a need for equipment and professional help, which they felt they had to battle the system to obtain: “everything you had to ask for basically”; “you should get that anyway without having to ask” [13].

Participants also expressed how the burden of the battle for support felt like it added to the difficulties of recovery: “it was something else I had to contend with on top of trying to get better” [13].

This was also expressed in another study as “I received quite a lot of information, I think mainly because I was very persistent” [26].

Participants separately identified the need for information and someone to coordinate advice, services, and care: “I’m not a doctor but I would like to know if I’m doing the right thing, or if I’m just going to make it worse” [26].

“A person whose job it is to follow up patients who have been critically ill and can link to any necessary services after discharge” [25] was very clearly picked up by one participant and supported by another in the same study: “a named person who I can contact and could help me and my family get my life back” [25].

Finally, the need for education arose from many findings, some of which have been linked to other categories, such as the need for healthcare professionals’ input, which was identified as a motivating factor in synthesised finding 1 (people experience changes in momentum of recovery). The need for education in this theme reflected feelings of uncertainty: “right now, I would like to know more about what happened…”; “no one talked to you, no one said he’s doing this, he’s doing that”; and “I had no idea what I was in for” [26]. Alongside uncertainty, there was recognition of the benefit of education: “the home care nurse reassured us that everything was going along as well as could be expected and she was just tremendous” [26].

Finally, one survivor illustrated the need for education to highlight the conflict between the reality and the appearance to others: “you can’t see it, so it’s easy to pretend it does not exist…. I don’t look like a disabled person who can hardly climb a staircase” [25].

## 4. Discussion

This review aimed to explore critical care survivors’ perceptions, opinions, and experiences of physical recovery and physical rehabilitation following hospital discharge. Three synthesised findings were identified from the categories and findings supported by evidence from within the included studies. These were “People experience challenges in momentum for physical recovery”; “Positivity, Motivation & Hope”, and “Recovery is Hard; People need Support”. All findings were unequivocally supported by the voices of participants or, where specifically relevant, the voices of relatives/carers in the studies. The ConQual summary of the findings (see Supplementary Material S2) identifies the strength of evidence as high or moderate for all areas. These synthesised findings highlighted the complex physical and emotional challenges influencing the physical recovery of critical illness survivors; the perceived value of professional expertise and structured rehabilitation programmes; and the lack of support and information made available.

The limited body of evidence identified by the review suggests that to date, there has been a lack of focus on these topics, despite widespread recognition of the importance of this area of practice [27] and the provision of related best practice guidelines [9]. In contrast, there have been many studies contributing to a significant body of evidence investigating interventions for rehabilitation within the hospital environment.

Although there is an increasing focus on maximizing recovery and rehabilitation following critical illness, there remains uncertainty regarding what should be delivered, and how. Studies testing single and bundled interventions have not shown a significant impact on medium- and long-term outcomes. They suggest the reason for this is unclear but may relate to the complex nature of care provision following critical illness. However, given the lack of qualitative data identified in this review, it is also possible that the limited focus on the voices of survivors to date has led to the development of services that healthcare professionals consider to be needed, rather than service design being driven by user input. Vollam and Efstathiou [28] suggest that considering the consequences for patients, family members, and service provision, understanding the long-term effects of critical illness, and maximizing physical recovery should be priorities for multi-professional clinicians.

The first objective of the review was to identify the positive and negative aspects of physical activity or physical rehabilitation interventions from the patient’s perspective. Prior to commencing this review, it was anticipated that more literature related to patients’ experiences of specific rehabilitation interventions would be retrieved. This, however, was not the case, with only two such studies being included [13,23]. Although the other studies in this review were focused on wider experiences of physical recovery and rehabilitation, there was little discussion of the impact of any specific interventions participants had received. From the synthesis of findings, it is not possible to conclude whether this is due to the lack of availability (or uptake) of services, or the lack of a perceived impact of the interventions that were available on the overall experience of physical recovery.

Given the paucity of evidence evaluating specific interventions, it is challenging to compare the findings of this review with the wider evidence base. However, the positive aspects of structured exercise that were identified are in agreement with the wider literature. For example, in a qualitative study of 20 ICU survivor caregivers based in the USA, the UK, and Australia, it is clear that their perception of interventions included the positive benefits of structured programmes. Our review also highlighted that programmes were seen by participants as a motivating factor in rehabilitation, affecting the intrinsic motivation for recovery and extrinsic drive for independence during the programmes.

Beyond perceptions of specific interventions, this review identified positive and negative aspects of survivor experiences of rehabilitation and physical recovery, which may have relevance for the development of future interventions. Some of the negative perceptions related to the challenges of changes in the momentum of recovery and emotional challenges, particularly frustration, anger, and despondency, were associated with changes in ability following illness. The importance of ‘momentum’ in recovery has been identified in other groups (for example, stroke survivors) as a significant factor supporting motivation leading to recommendations that post-stroke rehabilitation should support people to approach their recovery as a long-term trajectory, rather than a short-term ‘intervention’ [29]. Given the findings of this review, it is likely that these aspects may also be relevant for critical illness survivors.

The category “being a burden” illustrates that similar experiences were perceived differently by individuals. For example, whilst some participants were frustrated at being a burden due to reduced ability, in others, this experience created a motivation to improve and become more independent. As with other groups, this finding emphasises the importance of personalised care and tailoring of both rehabilitation content and approach [30].

Following on from this, the synthesised finding, “positivity, motivation and hope”, links closely with the importance of independence to survivors. The desire to regain independence and persevering or training to achieve this was a positive aspect of recovery and rehabilitation. This could also be seen in the context of regaining control that was lost during critical illness. Finally, the intrinsic motivation and hope in recovery were seen in findings where participants identified milestones in recovery and a positive outlook, summarised as “Hang in there… don’t give up”!

The second objective was to identify experiences relating to aspects of rehabilitation interventions such as the location, content, and frequency of service provision.

Although there was a clear expression of positivity around the structured exercise programmes, there was no overt discussion relating to aspects of provision such as location or organisational structure. In addition, the two studies in this review that evaluated specific interventions [13,26] had methodological quality ratings lower than those of the other five studies; therefore, it is important to apply relevant caution to the interpretation of the findings. Consequently, it is difficult to draw specific conclusions regarding this particular objective. It may be that these aspects of rehabilitation interventions have not been evaluated in a qualitative manner or that the lack of qualitative studies reflects the relatively sparse research in the area in general.

Participants’ experiences of specific programmes were considered by Parry and Knight [17] in their review of the factors influencing physical activity and rehabilitation in survivors of critical illness. This systematic review of qualitative and quantitative studies included eighty-nine papers; however, the majority of the included studies focussed on inpatients. Despite the differing focus of the review, one of the synthesised themes identified that structured inpatient physical activity programmes reduced boredom, improved motivation, psychological, and physical outcomes, and provided much-needed support for patients and carers. Despite the different settings, this finding does suggest that structured rehabilitation programmes post-discharge could have value in mitigating some of the negative experiences identified in our review.

Further research exploring the experiences and perceptions of formal interventions such as exercise programmes will add to the understanding of how survivors feel about these, but there is also a paucity of evidence around less formal interventions. These could include activities such as community rehabilitation, access to therapy via GP surgeries, and self-directed physical activity either with carers or family or in independently accessed classes.

The final objective of this review was to gain an understanding of the issues which influence patients’ experiences of physical recovery, either positively or negatively. This review identified that physical recovery was perceived to be negatively impacted by poor coordination of services, lack of information, and equipment.

A review by Smith and Lee [31] highlighted that the lack of information and poor coordination of services may be related to the lack of awareness of the issues for survivors amongst community-based healthcare professionals, which leaves them as an underserved population. This study focused on the impact of PICS in ICU COVID-19 survivors, highlighting the physical effects of PICS. Smith et al. [31] also identified the need for further research on the efficacy of interventions in order to prioritise rehabilitation interventions.

Whilst funding for the provision of post-discharge rehabilitation has been identified as a significant limitation to UK service provision [11], the findings of this review suggest that support for physical recovery needs to move beyond ‘simply’ providing survivors with opportunities to engage in structured exercise. For example, the strength of feeling around being discharged home without support, advice, or continuity of care was very evident, with participants expressing a range of feelings akin to abandonment. However, the ongoing involvement of healthcare professionals in areas such as goal setting and recovery planning was perceived to mitigate such experiences, at least in part. Worryingly, despite this being a relatively well-established area of research, and one where services have been developed and implemented [5], recent qualitative work suggests that many survivors continue to experience these psychological issues and still feel they lack information [32].

### Limitations of this Review

The inclusion criteria for this review specifically focused on qualitative studies involving participants who had been discharged from hospital, with the exclusion of studies where some hospital-based data were collected. This was to prevent this review from becoming focused on in-hospital perceptions and experiences, as with previous studies. However, this limited the number of studies that could be included and, therefore, may have missed some qualitative data that would be relevant to the post-discharge experience but had been expressed by those during admission.

The inclusion of only English-language studies could also have affected the range of data that were available for inclusion in this review.

## 5. Conclusions

This review has identified that the qualitative evidence around the experiences of physical recovery and rehabilitation interventions following discharge home after critical illness is limited.

The findings indicate that ICU survivors struggle to access services following discharge home, leaving them fighting for support, feeling abandoned, and wanting to know what is normal and expected for their recovery. There are also challenges in the momentum of physical recovery, which are compounded by physical weakness and frustration, emotional issues, and a lack of motivation.

There is an unmet need for support, information, and access to healthcare professionals for ICU survivors. This unmet need influences survivors’ physical recovery, but it is unclear whether this need would be met by a rehabilitation intervention or whether other services would be needed.

Positive influences on physical recovery included the expertise and support of healthcare professionals and the importance of independence. Supervised exercise programmes had a positive impact on the perception of recovery and motivation; however, it is likely that services will need to move beyond ‘simple’ structured exercise provision in order to meet the range of challenges experienced by ICU survivors. Whilst some factors influencing physical recovery are similar to other groups, there are unique issues experienced by those returning home after critical illness. Therefore, further qualitative research is needed to explore the needs of ICU survivors to regain their physical function and independence. Further research exploring the influences on their physical recovery and return to function would also inform the design of services and rehabilitation interventions.

## Figures and Tables

**Table 1:** Inclusion and exclusion criteria.

Inclusion Criteria	Exclusion Criteria
Qualitative studies	Quantitative studies
Studies conducted with participants discharged home after a critical care episode	Studies involving participants still in the hospital environment
Studies focused on perceptions/experiences/opinions of physical rehabilitation (interventions) or physical activity (in-tervention) after discharge home from hospital	Focus of study not on perceptions/experiences/opinions of physical rehabilitation or physical activity after discharge
English language	Not published in English language
Adult participants	Not focused on adult participants

Participants: studies using qualitative methodologies including adults over 18 years of age who had been discharged home following an intensive care episode and who expressed opinions and perceptions or discussed experiences of physical activity or physical rehabilitation intervention following hospital discharge.

## Data Availability

All data connected to this review are available within the online Supplementary Materials sections.

## Supplementary Materials

The following supporting information can be downloaded at https://www.mdpi.com/article/10.3390/nursrep14010013/s1. Supplementary Material S1: Table of included studies; Supplementary Material S2: ConQual summary of findings. Supplementary Material S3: JBI appraisal tool for qualitative research; Supplementary Material S4: Meta-aggregated flow charts for synthesised findings, categories, and findings.

## Public Involvement Statement

There was no public involvement in any aspect of this research.

## Guidelines and Standards Statement

This manuscript was drafted against the Preferred Reporting Items for Systematic Reviews and Meta-Analysis (PRISMA) [18].
