# Supplementary material for: The Experience of Physical Recovery and Physical Rehabilitation Following Hospital Discharge for Intensive Care Survivors—A Qualitative Systematic Review
Source: Nurs Rep. 2024 Jan 9;14(1):148–63. doi: 10.3390/nursrep14010013 (PMC10801540; doi:10.3390/nursrep14010013)
Supplement: Supplementary file 1 [file nursrep-14-00013-s001.zip › nursrep-2720619-supplementary.pdf]

The experience of physical recovery and physical rehabilitation following hospital discharge for intensive care survivors – a qualitative systematic review. **Sian Goddard [1], Rachel Dennet [1], Bridie Kent[2] and Hilary Gunn[1]**

Supplementary Material S1. Table of included studies.

Study	Methods for data collection and analysis	Country	Phenomena of interest	Setting/context/culture	Participant characteristics and sample size	Description of main results
Agård AS, Egerod I, Tønnesen E, Lomborg K. (2012) Struggling for independence: A grounded theory study on convalescence of ICU survivors 12 months post ICU discharge	semi-structured interviews at pt home or quiet room in hospital. The 60—90 minute interviews were audio-taped and transcribed verbatim. Also 2 focus groups patients and 2 focus groups partners. some participants d/c 12/12 previously, some 3/12 previously. Focus groups around 12/12 post d/c. Grounded theory analysis	Denmark	The aim of the study was to explore the challenges facing ICU survivors with a cohabiting spouse or partner and explain patients' concerns and coping modalities during the first 12 months post ICU discharge.	Five ICUs in Denmark,	n=36 (18 patient/18 participants), inclusion criteria (1) ICU survivors aged 25—70 years (people of working age), (2) intubation > 96 hours (to target the more severely ill patients) (Douglas and Daly, 2003), (3) patients with a cohabiting partner (potential primary caregiver after discharge), and (4) ability to communicate adequately in Danish. Patients were aged 35—70 years; 11 were men, 7 women. The ICU survivors, who were all generally in good health prior to critical illness, reported a wide range of complications in the first year after ICU discharge. The majority had experienced weight loss (5—25 kilos), fatigue, and loss of appetite. Two had persistent residual symptoms after critical illness polyneuropathy affecting their fine motor skills. Another patient had difficulty swallowing as a result of intubation. Depending on the ICU admission diagnosis, some of the patients also had various injuries causing reduced physical function (e.g. bone fractures)	18 patients and their partner at 3/12 post ICU d/c, One couple and one partner withdrew from the study before the 12-month interview (17patient/16partners at 12 months). focus group interviews with patients only (n = 3 and n = 7) and two with partners only (n = 2 and n = 7). Most of the patients with primary brain injuries or cerebral problems secondary to other conditions, e.g. hypoxia or septicaemia, reported cognitive symptoms such as reduced memory, concentration, or planning ability. Some patients with no known cerebral damage reported confusion or lack of initiative and reduced concentration or irritability in the initial period after ICU discharge and attributed these symptoms to their weak physical constitution in general. ICU survivors recalled unpleasant hallucinations or unreal experiences, while two others remembered pleasant hallucinations. Themes: Struggling for independence Recovering physical strength; Regaining functional capacity; Resuming domestic roles. Three phases

Study	Methods for data collection and analysis	Country	Phenomena of interest	Setting/context/culture	Participant characteristics and sample size	Description of main results
Corner EJ, Murray EJ, Brett SJ. (2019) Qualitative, grounded theory exploration of patients' experience of early mobilisation, rehabilitation and recovery after critical illness	semistructured interviews, Enrolment and interviews continued until thematic saturation was reached. interviews were anonymised, recorded and transcribed verbatim by a professional transcription company. Transcripts were uploaded onto NVivo software (QSR International, Doncaster, Australia) for analysis. Grounded theory approach to analysis	UK	To explore the patient experience of recovery from critical illness, with emphasis on their experience of rehabilitation.	adult medical/ surgical ICU of a 430-bedded London teaching hospital, United Kingdom	English speaking, a critical stay of >72 hours, capable of providing informed consent determined using the Mental Capacity Act assessment, anticipated to survive, aged over 18 years and documented intensive care unit acquired weakness (ICUAW) determined via case note review (this was to ensure that the participants had exposure to rehabilitation interventions). n=15 (plus 4 relatives present)	The median ICU and hospital length of stay were 19 days (IQR 8–33) and 63 days (IQR 34–107), respectively. The median time between ICU discharge and interview was 56 days (IQR: 36–80). Ten (66.6%) of the interviews took place at the hospital while the patients were still inpatients, and five (33.3%) took place after discharge in the patient's home (n=2), work (n=1) or in a clinic room (n=2). The interviews lasted a median of 39 min (IQR: 28–50). The central phenomenon grounded in these data was recalibration of the self. There were two themes contributing to this temporal model of recovery: the transition 'from prior self to current self' and the transition 'from current self to construction of the future self'.

Study	Methods for data collection and analysis	Country	Phenomena of interest	Setting/context/culture	Participant characteristics and sample size	Description of main results
Czerwonka AI, Herridge MS, Chan L, Chu LM, Matte A, Cameron JI. (2015) Changing support needs of survivors of complex critical illness and their family caregivers across the care continuum: a qualitative pilot study of Towards RECOVER.	In-depth semi structured qualitative interviews were conducted individually with either the survivor or caregiver. Framework analysis	Canada	The interview asked participants about support they received and desire for each phase of the adapted TIR framework. Also explored how they received support or how they would like to receive it in the future. Interviews conducted either in person/over telephone. Each participant interviewed multiple times during study with aim of having qualitative interviews parallel the timing of the quantitative assessments of the	conducted at 2 university-affiliated medical-surgical ICUs in Canada. Post ICU patients (interviewed approximately 7 days, 3, 6, 12, and 24 months post-ICU discharge).	n=12 - 5 survivors and 7 family caregivers. Survivors were eligible for enrollment if they were at least 16 years of age and mechanically ventilated for a minimum of 7 days. Survivors were excluded if they were non-ambulatory before ICU admission; had a catastrophic neurologic injury in the opinion of the attending intensivist; or had a preexisting neuromuscular disease or history of psychiatric admission. We included family caregivers who were primarily responsible for providing and/or coordinating any necessary assistance to the survivor without financial compensation and were at least 18 years of age. All participants had to read and speak English. We recruited a convenience sample from the Towards RECOVER pilot cohort to participate in the qualitative interviews. We interviewed caregiver-survivor dyads as well as survivors who did not have caregivers and caregivers whose survivor was not able or interested in participating.	Length of ICU stay= 24 days (10-61) 29 days (caregivers relative) (10-64). n=2 (ICU survivors and n=2 caregiver relatives) attended IP rehab Interviews conducted time post-ICU discharge: 7 days, 1 interview etc (see table 2 of paper pg 244 Overriding theme: Survivors do not experience continuity of medical care during recovery after critical illness. Subtheme 1: Informational needs change across the care continuum Subtheme 2: Fear and worry exist when families do not know what to expect Subtheme 3: Survivors transition from dependence to independence Future research should focus on understanding and easing transitions over the long term by giving survivors and caregivers the information and support they need across the various phases of recovery, especially 1 to 2 years post discharge when it is most lacking.More research on how family behaviors can affect patient independence and recovery should be conducted to develop interventions that ease this process.

Study	Methods for data collection and analysis	Country	Phenomena of interest	Setting/context/culture	Participant characteristics and sample size	Description of main results
Deacon KS. (2012) Re-building life after ICU: A qualitative study of the patients' perspective	web-based qualitative study that sought to explore patients' experiences using an open-ended questionnaire (online survey). Thematic approach to analysis guided by principles of grounded theory	UK led but responses from UK, USA, Australia, Canada	The current study aimed to explore former patients' views on the key components of a post ICU rehabilitation programme. The primary research question was: (1) What did ex-ICU patients feel were the key components of a rehabilitation programme following discharge from ICU? Subsidiary questions were: (2) How did participants experience being a patient in ICU? (3) How did participants experience the	Participants had been ICU patients ? how long ago	n=35 Participant ages ranged from 22 to 70 years with a mean age of 48 (SD — 9.79). There were 30 females and five males. Participants were from a variety of countries, predominantly the United States of America (USA) (22) and the UK (8), but also Canada (2) and Australia (1).Two participants did not give their nationality. Length of stay (LOS) in ICU ranged from four days to four months with a mean of 36.3 days (SD — 28.3). Experience of post ICU was variable.	The relationship of each theme to the participants and to the subthemes was developed into a three way model of rehabilitation needs- Information and education, Assessment and therapy, Personal support

Study	Methods for data collection and analysis	Country	Phenomena of interest	Setting/context/culture	Participant characteristics and sample size	Description of main results
Ferguson K, Bradley JM, McAuley DF, Blackwood B, O'Neill B. (2019) Patients' Perceptions of an Exercise Program Delivered Following Discharge From Hospital After Critical Illness (the Revive Trial)	semi-structured interviews. Interviews were audio recorded, transcribed and content analysis used to explore themes arising from the data. Interviews were face-to-face interview (14), or via telephone if necessary (7), at their 6 month follow-up time point	Northern Ireland	explore patients' satisfaction with the exercise programme, perceptions of physical and psychological effects of the programme, views about continuing exercise, and perceptions of the outcome measures used in the trial. (individually tailored (personalised) 6 week exercise programme for patients discharged from hospital after critical illness (The REVIVE trial).	general intensive care units in six hospitals in Northern Ireland.	n=21 participants who had completed the REVIVE trial exercise (see main revive trial for inclusion criteria) doi:10.1136/thoraxjnl-2016-208723. Eligible patients were aged ≥18 years, had received mechanical ventilation for >96 hours, were planned to be discharged home, were medically fit to participate and were not participating in another rehabilitation programme, that is, cardiac rehabilitation or pulmonary rehabilitation	Interviews were completed face-to-face by 14 (67%) patients, and 7 (33%) by phone call. Interviews lasted an average of 25 minutes per patient. Two patients requested their spouses to be present during the interview and they contributed data to the interviews. participant mean age was 53 (13) years. 48% male with mean ventilation 311.0 (308.9) hours. Core theme 1: Sequelae of critical illness and critical care recovery Core theme 2: Satisfaction and endorsement of the exercise programme Core theme 3: Beneficial impact of the exercise programme on physical and psychological health Core theme 4: Facilitators of beneficial impact Core theme 5: Barriers to beneficial impact Core theme 6: Challenges to continuing exercise after the 6-week programme Core theme 7: Contrasting views on outcome measures
Redwine A. (2007) Perceptions and abilities over the rehabilitation trajectory: A study of post ICU recovery.	Interviews and framework analysis.	USA	Older people following critical care episode	Various community settings inlcuding homes and nursing homes	Ten participants aged between 65 and 97. Equal numbers of males/females. Recruitment was via links with chaplaincy teams or local ministers, not via hospital or community staff.Each participant was interviewed five times between one week and 24 weeks post discharge home.	Five themes established through comparative analysis. a) Mismatched Assumptions and Experienced Realities; b) Fluidity of Perceptions; c) Uncertainty/Fear Related to the Unknown; d) Social Comparison/Concept of Normal; and, e) Religion used as a Mechanism of Coping and Adaptation.

Study	Methods for data collection and analysis	Country	Phenomena of interest	Setting/context/culture	Participant characteristics and sample size	Description of main results
Walker W, Wright J, Danjoux G, Howell SJ, Martin D, Bonner S. (2015) Project Post Intensive Care eXercise (PIX): a qualitative exploration of intensive care unit survivors' perceptions of quality of life post-discharge and experience of exercise	focus groups, audio recorded, transcribed. Thematic analysis as described by Braun and Clarke	UK	explore in more detail recovery from critical illness, quality of life following hospital discharge, perceptions of the (PIX) exercise programme (8 week in-hospital supervised exercise programme,) and it's impact on perceived well-being. The main questions were: How has your stay in ICU affected your quality of life following discharge? Have you any comments about the aftercare you received or would have liked to receive? What	D/c home post ICU from two large teaching hospitals	n=16 purposive sampling to include equal representation of men and women of different ages, exercise and control groups and those with a diagnosis of trauma and sepsis. for survivors of critical illness (minimum of 3 days ventilation). Patients admitted to the ICU of one of two large teaching hospitals were invited to participate. Eligible patients were aged 18–65 yr, had received a minimum of 3 days of ventilator support (for the emergency management of trauma or sepsis), and had been discharged home within 6 months of hospital admission. The study exclusion criteria were the inability to climb a flight of stairs, enrolment in another rehabilitation programme, and medical contraindication to cardiopulmonary exercise testing.10 Patients were recruited either before discharge from hospital or in the ICU follow-up clinic.	Sixteen participants (eight of whom underwent the supervised exercise programme) were allocated to four psychologist lead focus groups. Mean age 42.6 (14.8) years, days ventilated 14 (10-23), 70% male. Significant biopsychosocial adjustment process post-ICU Negative and enduring physical effects impacting Negative psychological effects Social withdrawal Boredom/inactivity Positive psychological effects Negative experiences of community aftercare Feeling abandoned/uncared for Lack of advice/information for self and families Delays before outpatient physiotherapy Battling the system Positive biopsychosocial effects of the exercise programme External source of motivation Reduced boredom, isolation and inactivity Intrinsically enjoyable Positive recovery focus enhanced well-being Feeling cared about/emotional support from staff Improved fitness Accessible form of exercise Suggestions for better aftercare Group exercise/physiotherapy to enhance motivation

Supplementary Material S2. ConQual Summary of Findings

Title: The experience of physical recovery and physical rehabilitation following hospital discharge for intensive care survivors – a qualitative systematic

Synthesised Findings	Type of research	Dependability	Credibility	Conqual Score	Comments
			All findings were unequivocal		
**Barriers**					
Burden	Qualitative	Unchanged	Unchanged	High	
Weakness	Qualitative	At least one paper scored 'Yes' for 3 of 5 questions; so downgrade to moderate.	Unchanged	Moderate	2 of 4 findings from moderate dependability scoring papers
Boredom	Qualitative	At least one paper scored 'Yes' for 3 of 5 questions; so downgrade to moderate.	Unchanged	Moderate	Findings from moderate scoring papers for dependability
Emotions	Qualitative	At least one paper scored 'Yes' for 3 of 5 questions; so downgrade to moderate.	Unchanged	Moderate	4 of 5 findings from moderate scoring papers for dependability
Lack of motivation	Qualitative	At least one paper scored 'Yes' for 3 of 5 questions; so downgrade to moderate.	Unchanged	Moderate	1 of 3 findings from moderate scroing papers for dependability
**Motivation**					
Healthcare professionals	Qualitative	At least one paper scored 'Yes' for 3 of 5 questions; so downgrade to moderate.	Unchanged	Moderate	2 of 7 findings from moderate scoring papers for dependability
Positivity	Qualitative	At least one paper scored 'Yes' for 3 of 5 questions; so downgrade to moderate.	Unchanged	Moderate	2 of 5 findings from moderate scoring papers for dependability
Independence	Qualitative	Unchanged	Unchanged	High	
Ex programme	Qualitative	At least one paper scored 'Yes' for 3 of 5 questions; so downgrade to moderate.	Unchanged	Moderate	All 11 findings from moderate scoring papers for dependability
**Need for Support**					
Need for education	Qualitative	Unchanged	Unchanged	High	
Abandoned	Qualitative	At least one paper scored 'Yes' for 3 of 5 questions; so downgrade to moderate.	Unchanged	Moderate	Both findings from moderate scoring papers for dependability
Fighting for support	Qualitative	At least one paper scored 'Yes' for 3 of 5 questions; so downgrade to moderate.	Unchanged	Moderate	1 of 2 findings from moderate scoring papers for dependability
What is normal and expected	Qualitative	Unchanged	Unchanged	High	
Co-ordinator for care or information	Qualitative	Unchanged	Unchanged	High	

JBI ConQual Summary of Findings

Title: The experience of physical recovery and physical rehabilitation following hospital discharge for intensive care survivors – a qualitative systematic review.

**Population: describe population of interest**	Survivors of critical illness who have been discharged home
**Phenomena of interest: insert the specific phenomena of interest**	Studies investigating the participants experiences or perceptions of physical recovery or rehabilitation following critical illness
**Context: Concise description of the key contextual factors**	Studies involving participants who had been discharged home from hiospital and were involved in qualitative research considering their physical recovery or rehabilitation

Table: Qualitative Research

Citation	Q2	Q3	Q4	Q6	Q7	Score - Qualitative	Dependability score	Dependability Level	Score	Credibility for findings within the paper	ConQual Score
	2. Is there congruity between the research methodology and the research question or objectives?	3. Is there congruity between the research methodology and the methods used to collect data?	4. Is there congruity between the research methodology and the representation and analysis of data?	6. Is there a statement locating the researcher culturally or theoretically?	7. Is the influence of the researcher on the research, and vice-versa, addressed?						
Agård AS, Egerod I, Tønnesen E, Lomborg K. (2012) Struggling for independence: A grounded theory study on convalescence of ICU survivors 12 months post ICU discharge	Y	Y	Y	Y	N	High	4	Unchanged	High	Unequivocal	High
Corner EJ, Murray EJ, Brett SJ. (2019) Qualitative, grounded theory exploration of patients' experience of early mobilisation, rehabilitation and recovery after critical illness	Y	Y	Y	Y	Y	High	5	Unchanged	High	Unequivocal	High
Czerwonka AI, Herridge MS, Chan L, Chu LM, Matte A, Cameron JI. (2015) Changing support needs of survivors of complex critical illness and their family caregivers across the care continuum: a qualitative pilot study of Towards RECOVER.	Y	Y	Y	Y	N	High	4	Unchanged	High	Unequivocal	High
Deacon KS. (2012) Re-building life after ICU: A qualitative study of the patients' perspective	Y	Y	Y	N	Y	High	4	Unchanged	High	Unequivocal	High
Ferguson K, Bradley JM, McAuley DF, Blackwood B, O'Neill B. (2019) Patients' Perceptions of an Exercise Program Delivered Following Discharge From Hospital After Critical Illness (the Revive Trial)	Y	Y	Y	N	N	High	3	Downgrade 1 level	Moderate	Unequivocal	Moderate
Redwine A. (2007) Perceptions and abilities over the rehabilitation trajectory: A study of post ICU recovery.	Y	Y	Y	Y	U	High	4	Unchanged	HIgh	Unequivocal	HIgh
Walker W, Wright J, Danjoux G, Howell SJ, Martin D, Bonner S. (2015) Project Post Intensive Care eXercise (PIX): a qualitative exploration of intensive care unit survivors' perceptions of quality of life post-discharge and experience of exercise	Y	Y	Y	N	N	High	3	Downgrade 1 level	Moderate	Unequivocal	Moderate

Supplementary Material S3. JBI critical appraisal tool for Qualitative Research.

JBI Critical Appraisal Checklist for Qualitative Research

Reviewer_________________________________Date_________________________

Author _________________________________Year_________Record Number______

	Yes	No	Unclear	Not applicable
1. Is there congruity between the stated philosophical perspective and the research methodology?	☐	☐	☐	☐
2. Is there congruity between the research methodology and the research question or objectives?	☐	☐	☐	☐
3. Is there congruity between the research methodology and the methods used to collect data?	☐	☐	☐	☐
4. Is there congruity between the research methodology and the representation and analysis of data?	☐	☐	☐	☐
5. Is there congruity between the research methodology and the interpretation of results?	☐	☐	☐	☐
6. Is there a statement locating the researcher culturally or theoretically?	☐	☐	☐	☐
7. Is the influence of the researcher on the research, and vice- versa, addressed?	☐	☐	☐	☐
8. Are participants, and their voices, adequately represented?	☐	☐	☐	☐
9. Is the research ethical according to current criteria or, for recent studies, and is there evidence of ethical approval by an appropriate body?	☐	☐	☐	☐
10. Do the conclusions drawn in the research report flow from the analysis, or interpretation, of the data?	☐	☐	☐	☐

Overall appraisal: Include ☐ Exclude ☐ Seek further info ☐

Comments (Including reason for exclusion)

____

____

____

JBI Critical Appraisal Results for Included Studies										
Citation	Q1	Q2	Q3	Q4	Q5	Q6	Q7	Q8	Q9	Q10
Agård AS, Egerod I, Tønnesen E, et al. 2012.	Y	Y	Y	Y	Y	Y	N	Y	Y	Y
Corner EJ, Murray EJ, Brett SJ. 2019.	Y	Y	Y	Y	Y	Y	Y	Y	Y	Y
Czerwonka AI, Herridge MS, Chan L, Chu LM, Matte A, Cameron JI. 2015.	U	Y	Y	Y	Y	Y	N	Y	Y	Y
Deacon KS. 2012.	U	Y	Y	Y	Y	N	Y	U	U	Y
Ferguson K, Bradley JM, McAuley DF, Blackwood B, O'Neill B. 2019.	U	Y	Y	Y	Y	N	N	U	Y	Y
Redwine A. 2007.	Y	Y	Y	Y	Y	Y	U	Y	U	Y
Walker W, Wright J, Danjoux G, Howell SJ, Martin D, Bonner S. 2015.	U	Y	Y	Y	Y	N	N	U	Y	Y
%	42.85	100.0	100.0	100.0	100.0	57.14	28.57	57.14	71.42	100.0

Supplementary material S4: Synthesised findings, Categories, and findings.

Synthesised Finding 1: People experience challenges in momentum for physical recovery. Meta-aggregated flow chart of findings, categories, and synthesised finding.

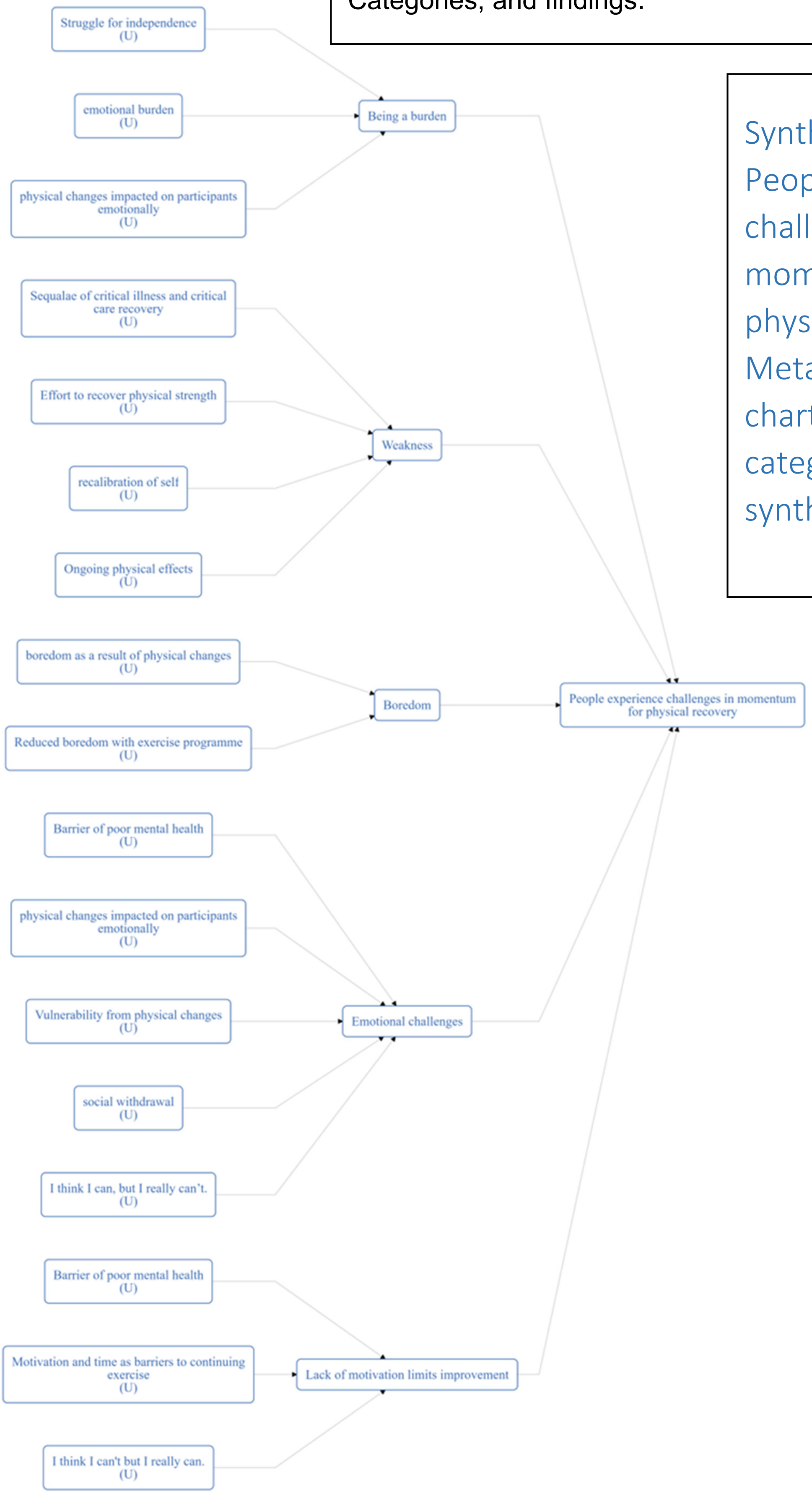

Synthesised Finding 2: Positivity, Motivation and Hope. Meta-aggregated flow chart of findings, categories and synthesised finding.

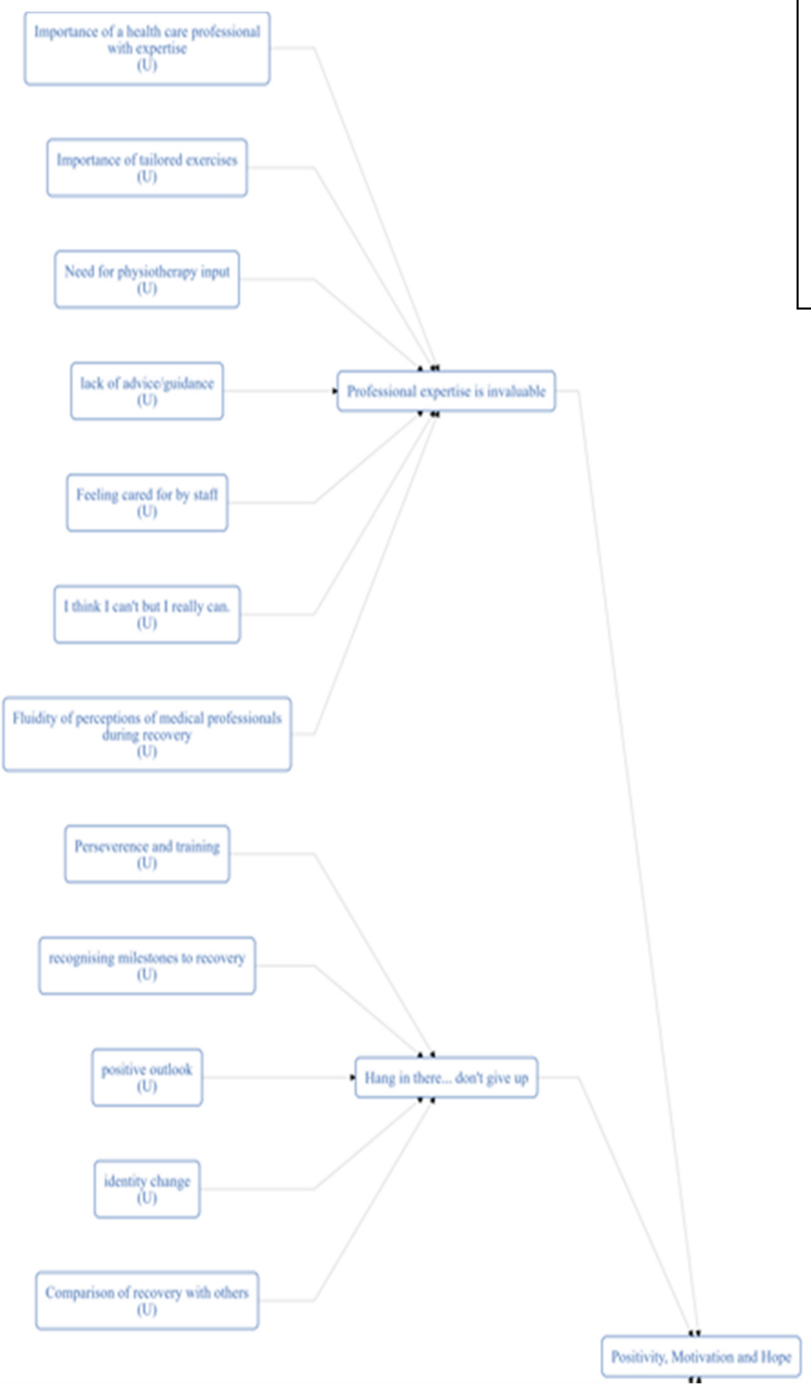

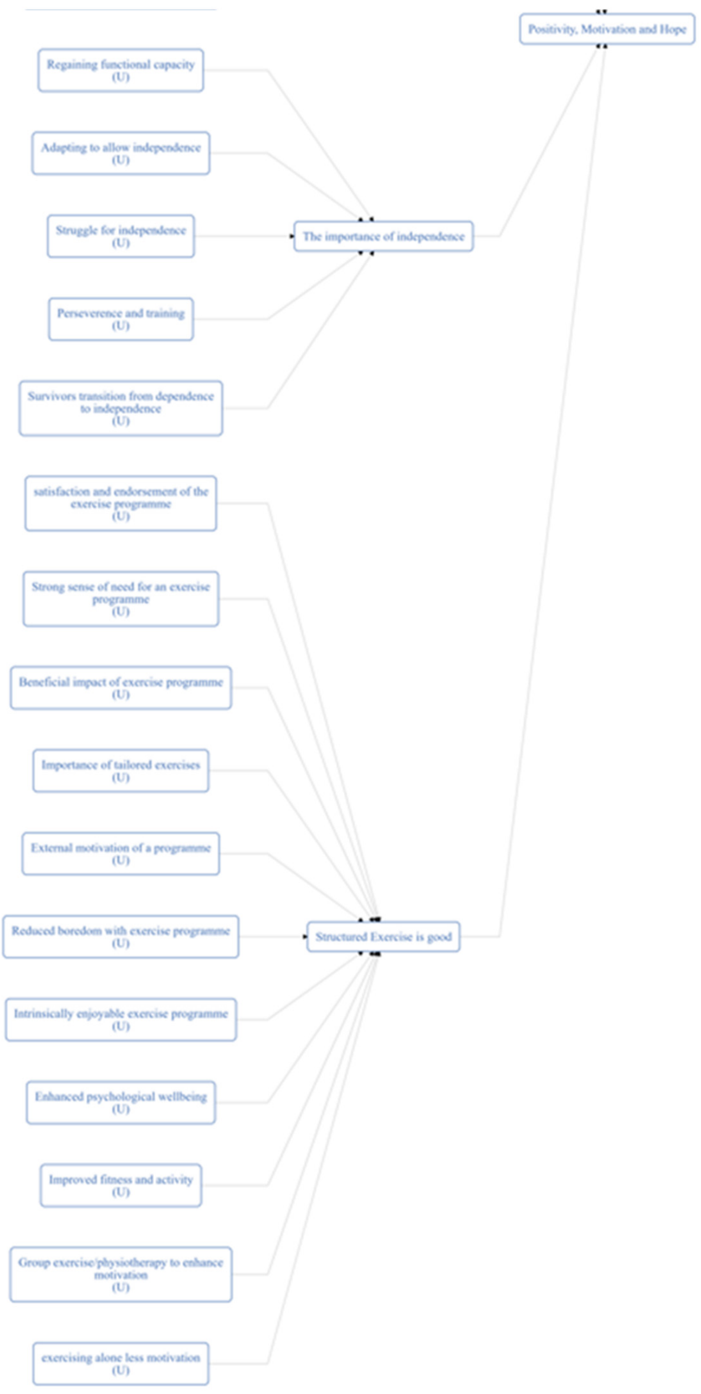

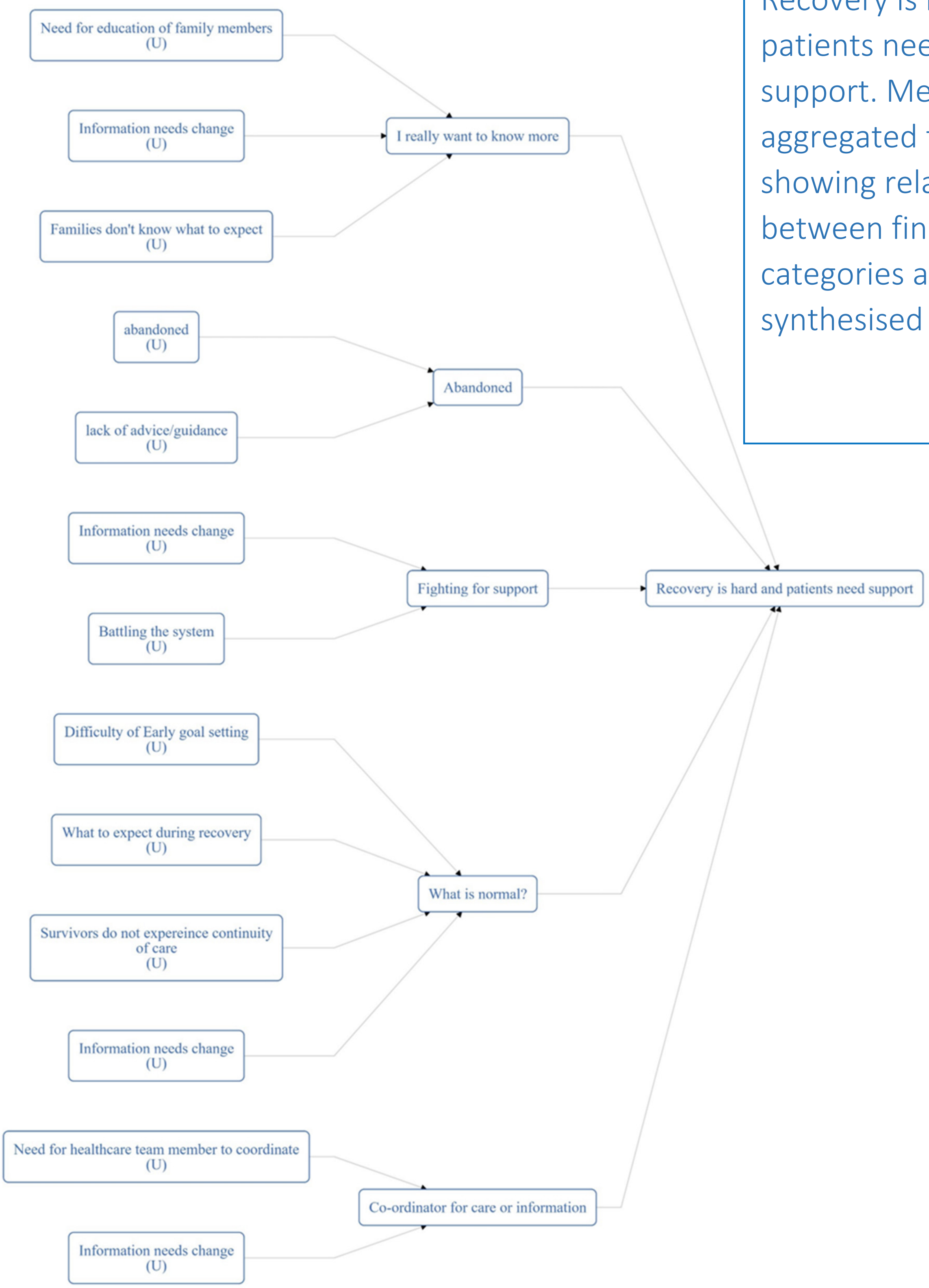

Synthesised finding 3: Recovery is hard and patients need support. Meta-aggregated flow chart showing relationships between findings, categories and the synthesised finding.
